# Effect of Lipid Additives and Drug on the Rheological Properties of Molten Paraffin Wax, Degree of Surface Drug Coating, and Drug Release in Spray-Congealed Microparticles

**DOI:** 10.3390/pharmaceutics10030075

**Published:** 2018-06-26

**Authors:** Hongyi Ouyang, Audrey Yi Zheng, Paul Wan Sia Heng, Lai Wah Chan

**Affiliations:** GEA-NUS Pharmaceutical Processing Research Laboratory, Department of Pharmacy, National University of Singapore, 18 Science Drive 4, Singapore 117543, Singapore; e0002153@u.nus.edu (H.O.); audreyzhengyi@u.nus.edu (A.Y.Z.); paulheng@nus.edu.sg (P.W.S.H.)

**Keywords:** paraffin wax, spray congealing, microparticle, lipid, viscosity, paracetamol

## Abstract

Paraffin wax is potentially useful for producing spray-congealed drug-loaded microparticles with sustained-release and taste-masking properties. To date, there is little information about the effects of blending lipids with paraffin wax on the melt viscosity. In addition, drug particles may not be entirely coated by the paraffin wax matrix. In this study, drug-loaded paraffin wax microparticles were produced by spray-congealing, and the effects of lipid additives on the microparticle production were investigated. The influence of lipid additives (stearic acid, cetyl alcohol, or cetyl esters) and drug (paracetamol) on the rheological properties of paraffin wax were elucidated. Fourier transform-infrared spectroscopy was conducted to investigate the interactions between the blend constituents. Selected formulations were spray-congealed, and the microparticles produced were characterized for their size, drug content, degree of surface drug coating, and drug release. The viscosity of wax-lipid blends was found to be mostly lower than the weighted viscosity when interactions occurred between the blend constituents. Molten paraffin wax exhibited Newtonian flow, which was transformed to plastic flow by paracetamol and pseudoplastic flow by the lipid additive. The viscosity was decreased with lipid added. Compared to plain wax, wax-lipid blends produced smaller spray-congealed microparticles. Drug content remained high. Degree of surface drug coating and drug release were also higher. The lipid additives altered the rheological properties and hydrophobicity of the melt and are useful for modifying the microparticle properties.

## 1. Introduction

**Figure 1:**
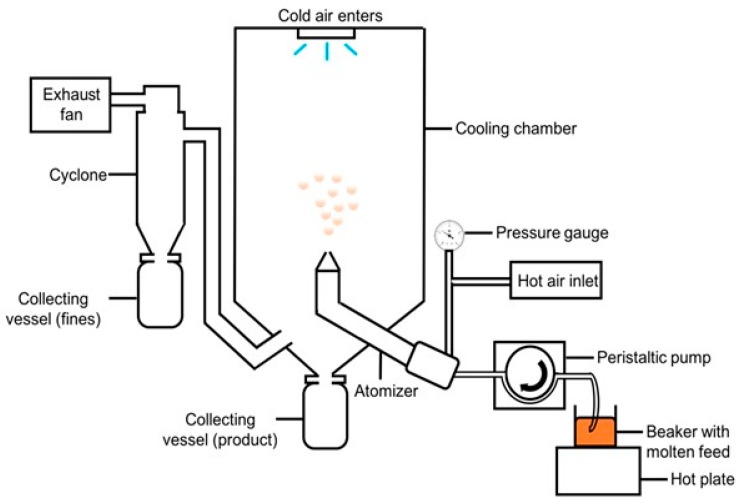
A schematic diagram of a laboratory scale spray congealer (counter-current set-up).

Spray congealing is a technique where a molten mixture is atomized into a chilled chamber in which the molten droplets rapidly congeal into solid microparticles. The molten mixture may contain one or more drugs that can be melted, dispersed, or dissolved within a matrix material. The set-up of a laboratory scale spray congealer is depicted in Figure 1. Spray congealing is a rapid, single-step process suitable for the production of spherical and discrete microparticles [1,2,3,4,5,6,7]. The process does not involve water [8,9], which makes it appealing for moisture-sensitive drugs. In addition, spray-congealed microparticles generally have high encapsulation efficiencies [1,10,11,12] and thus result in minimal drug loss and significant cost savings, especially for expensive drugs.

Spray congealing has been employed as a method of microencapsulation for a wide range of pharmaceuticals, foods, and flavors [13,14,15]. It can also be adapted for the production of specialized drug delivery systems [16]. Since spray-congealed microparticles consist of drug(s) embedded in or surrounded by a matrix material, they may be employed to enhance stability [8,17,18,19,20,21], improve flow, and mask unpleasant taste of drugs [1,13,22,23,24,25,26,27,28], as well as alter drug release profiles [2,6,13,29,30,31,32,33,34,35,36,37,38,39]. Drug release rates can be altered by a prudent choice of matrix materials [22], additives, and physical properties of the drug such as particle size and crystallinity [31]. Hydrophilic materials such as higher molecular weight polyethylene glycols [40,41,42], poloxamers [2,6,43] and gelucires [44,45,46] have been investigated as potential matrix materials to enhance the dissolution rates of poorly water-soluble drugs. On the contrary, by employing lipophilic materials such as carnauba wax [3], microcrystalline wax [34], hydrogenated vegetable oils [47,48,49], tristearin [50], stearic acid [22,51,52], glyceryl behenate [53] and glyceryl dibehenate [52,54], microparticles with sustained-release properties can be produced.

Viscosity, a fundamental physicochemical property affecting spray congealing [1,17,33,55,56], is the measure of a material’s resistance to flow. In spray congealing, viscosity of the molten feed is influenced by several factors such as the nature of the matrix material, the amount of drug or additive used, and the atomization air temperature. It is a critical attribute for deciding the viability of spray congealing and the properties of the microparticles produced. Melt viscosity will affect molten droplet size during the atomization process, which will consequently determine the final microparticle size [6,8]. Spray congealing is not suitable for highly viscous molten mixtures as they will not flow well and may clog the feed tube or atomizer, resulting in premature termination of the process [6]. Certain drugs increase the viscosity of the molten feed and previous observations have shown that it is difficult to achieve a drug loading exceeding 20% *w*/*w* in spray congealing with lipids [1].

Paraffin wax is traditionally derived from crude oil by a high-pressure hydrogenation process to produce a natural blend of n-alkanes, iso-alkanes, and cyclo-alkanes. It is not widely used in pharmaceutical dosage forms due to concerns about its quality. It was not readily available in pharmaceutical grade until recently, when the Fischer-Tropsch (FT) process was harnessed to produce long-chain hydrocarbons (CxHy) from syngas. [57] The product obtained was further subjected to distillation and hydrogenation to yield paraffin wax of a specific chain length. Being chemically defined with low iso-alkane content and very linear molecular structure, this synthetic material will have consistent and predictable properties. Besides, it is inert and stable to heat, making it a suitable candidate for spray congealing in the development of drug delivery systems.

Little attention has been paid to the potential of paraffin wax in the development of spray-congealed microparticles for taste masking and sustained release of drugs. As mentioned earlier, the viscosity of the molten feed is an important consideration in spray congealing because it determines the process feasibility and properties of the microparticles produced. Moreover, hydrophilic drugs do not have great affinity for hydrophobic matrix material, and studies in spray congealing have reported that the drug particle may not be entirely coated by the matrix material [11,34,45]. It is thus anticipated that the drug particles at the surface of paraffin wax microparticles may not be properly coated, which will affect the taste masking and sustained release of the drug. In the development of drug delivery systems, various additives may be included to achieve the desired properties. Hence, this study aimed to investigate the rheological properties of paraffin wax and the impact of drug and various lipid additives on this critical attribute in spray congealing. The influence of these components on the degree of surface drug coating and drug release was also studied to further assess the potential of paraffin wax as a matrix material for taste masking and sustained release of drugs.

## 2. Materials and Methods

### 2.1. Materials

Two types of paraffin wax (Sasol, Hamburg, Germany), Sasolwax 5803 (PW59) and Sasolwax EXP 1581 (PW84) with melting points 59 and 84 °C respectively, were used. Triple pressed stearic acid (SA; Timur Oleochemicals, Port Klang, Malaysia), cetyl alcohol (CA; Crodacol C90, Croda, Singapore), and cetyl esters (CE; Crodamol SS, Croda, Singapore) were used as lipid additives. Their corresponding melting points are 59, 50 and 44 °C The aqueous solubility of SA and CA are 0.597 and 0.041 mg/L at 25 °C respectively, whereas CE is practically insoluble in water.

Paracetamol (Granules India Limited, Hyderabad, India) was used as the model drug for taste masking. It exists as a crystalline powder with a melting point of 168 °C and is relatively soluble in water (14 g/L at 25 °C) but forms a suspension with molten paraffin wax.

A phosphate buffer (pH 7.4) was selected to mimic the fluid in the oral cavity. Potassium dihydrogen phosphate dihydrate, disodium hydrogen phosphate, potassium chloride, sodium chloride and deionized water were used to prepare the buffer. Sodium lauryl sulphate (BDH Chemicals Limited, Poole, UK) was used as surfactant in the drug release study.

### 2.2. Preparation of Melts for Rheological Tests and Spray Congealing

The melts consisted of single material or a blend of two materials. The required amounts of each material were accurately weighed into a beaker and transferred into a water bath maintained at 15 °C above the melting point of the single material or the higher melting point of the two materials in the blend.

### 2.3. Preparation of Samples for Fourier Transform-Infrared (FTIR) Spectroscopy

Individual materials were ground to form a powder for analysis. For blends, the required amounts of each material were accurately weighed into a beaker and transferred to a water bath maintained at 15 °C above the higher melting point of the two materials and stirred for 5 min. The blend was solidified in the refrigerator and ground to form a powder for analysis.

### 2.4. Rheological Tests

#### 2.4.1. Continuous Ramping Tests

Continuous ramping tests were conducted using a rheometer (AR-G2, TA Instruments, New Castle, DE, USA) with a parallel plate system (40 mm diameter, gap 200 µm) to determine the viscosity of the molten formulations at different shear stress. The viscosity of the individual materials (PW59, PW84, SA, CA, or CE) and blends (PW59 with SA, CA, or CE, and PW84 with PW59, SA, CA, or CE) in various proportions, namely 1:9, 3:7, 5:5, 7:3, and 9:1, were measured. The samples were first heated to a temperature of 10 °C above their peak melting temperatures and allowed to equilibrate for 2 min at this particular temperature. Then, the rheograms were obtained as the samples were sheared at an increasing shear stress from 0 to 10 Pa over a duration of 5 min. The viscosity was obtained from the slope of the rheograms for materials that showed Newtonian flow. This was calculated as the reciprocal of the gradient from the line of best fit using the last 25 data points, which were more representative of the system at equilibrium.

#### 2.4.2. Temperature Ramping Tests

Temperature ramping tests were conducted using a rheometer (AR-G2, TA Instruments, New Castle, DE, USA) with a parallel plate system (40 mm diameter, gap 200 µm) to investigate the viscosity–temperature relationship of the different formulations. It involved the application of a constant shear stress while ramping the temperature at a user defined rate. The samples were heated from their respective peak melting temperatures to 110 °C (blends without PW84) or 140 °C (blends with PW84) at a fixed shear stress of 5 Pa over a duration of 5 min. Viscosity values were noted at different time points over an increasing temperature. Temperature ramping curves of viscosity against temperature were then obtained.

### 2.5. FTIR Spectroscopy

FTIR spectroscopy (Spectrum 100, Perkin Elmer, Waltham, MA, USA) was used to investigate possible interactions between paraffin wax and lipid additives to explain the rheological properties of the blends. The attenuated total reflection method was employed. Before each sample was examined, the prism surface was cleaned with ethanol and dried. For the first measurement, a background reading was taken before the sample was positioned on the prism surface and compressed. Infrared spectra of the individual materials and blends were determined and subjected to analysis.

### 2.6. Rheological Tests with Drug

Continuous ramping tests were performed using a rheometer (AR-G2, TA Instruments, New Castle, DE, USA) with a parallel plate system (20 mm diameter) to ascertain the viscosity of the molten formulations at various shear stress. The effect of lipid additives on the rheological properties of drug-loaded paraffin wax and blends was investigated. Tests were conducted on PW59 and blends of PW59 with SA, CA, or CE in a 1:1 ratio containing paracetamol. The samples were heated to 69 °C which was 10 °C above their peak melting temperatures, and allowed to equilibrate for 1 min at this particular temperature. Then, the rheograms were obtained as the samples were sheared at an increasing shear stress over a duration of 5 min. The gap and shear stress used were first optimized for each sample.

### 2.7. Thermal Analysis

The thermal properties of the individual materials, physical mixtures and spray-congealed microparticles were ascertained using a differential scanning calorimeter (DSC-60, Shimadzu, Kyoto, Japan). A hermetically sealed aluminum pan was filled with 4 mg of sample and placed in the DSC furnace. It was then heated from 25 to 200 °C at a rate of 5 °C/min. An empty sealed aluminum pan served as a reference. The analyses were conducted in triplicates, and the results were averaged.

### 2.8. Production of Spray-Congealed Microparticles

Four formulations containing paracetamol were selected for spray congealing: PW59 and blends of PW59 with SA, CA, or CE in a 1:1 ratio. A laboratory scale spray congealer (Mobile Minor 2000, GEA-Niro, Søborg, Denmark) was used. It consisted of a cylindrical chamber (internal diameter: 0.8 m, height: 0.86 m) with a conical base. A pneumatic fountain two-fluid nozzle equipped with a 2.0 mm nozzle tip was employed for the atomization of the molten material. An atomizing pressure of 0.3 bar was used with the cooling chamber maintained at 10 °C Atomization air temperature was maintained at 10 °C above the melting point of PW59. The molten material was maintained at 5 °C above the atomization air temperature using a water bath and conveyed to the spray nozzle via a peristaltic pump (Masterflex, Cole-Palmer, Vernon Hills, IL, USA) at a rate of 50 mL/min. The useful fraction of the spray-congealed microparticles was collected in a bottle at the bottom of the chamber. Fines (<10 µm) were entrained from the chamber along with the exhaust air and collected in another bottle at the cyclone.

### 2.9. Determination of Useful Yield and Total Yield of Spray-Congealed Microparticles

The microparticles produced were divided into two fractions. The useful fraction was obtained from the bottle at the bottom of the chamber and the fines was obtained from the bottle at the cyclone. The useful yield and total yield were calculated as follows:

(1) $$\mathrm{Useful}\;\mathrm{yield}\;\left(\%\right)=\;\frac{\mathrm{Weight}\;\mathrm{of}\;\mathrm{useful}\;\mathrm{fraction}}{\mathrm{Weight}\;\mathrm{of}\;\mathrm{starting}\;\mathrm{material}}\;\times \;100$$

(2) $$\mathrm{Total}\;\mathrm{yield}\;\left(\%\right)=\frac{\mathrm{Weight}\;\mathrm{of}\;\mathrm{useful}\;\mathrm{fraction}+\mathrm{weight}\;\mathrm{of}\;\mathrm{fines}}{\mathrm{Weight}\;\mathrm{of}\;\mathrm{starting}\;\mathrm{material}}\;\times \;100$$

The weight of the starting material was calculated by subtracting the weight of the container of molten mixture after spray congealing from the weight before the process.

### 2.10. Surface Examination of Spray-Congealed Microparticles

The morphology of pure paracetamol and spray-congealed microparticles was visualized using a scanning electron microscope (JSM-6010LV, JEOL, Tokyo, Japan). The samples were mounted on aluminum studs using conductive carbon tape before they were sputter coated with platinum using a magnetron sputter coater (MSP-2S, IXRF Systems, Austin, TX, USA) and observed at 5.0 kV under vacuum.

### 2.11. Determination of Drug Content

The sample (100 mg) was weighed and transferred into a 100 mL volumetric flask. It was then topped up to volume with phosphate buffer, and the flask was placed in a shaker bath (Precision Model 50, Thermo Scientific, Waltham, MA, USA) at 69 °C and agitated at 60 oscillations per min for 1 h. The mixture was then cooled to room temperature before an aliquot was taken through a 0.45 µm filter membrane (RC, Sartorius, Göttingen, Germany). Spectrophotometric analysis (U-5100, Hitachi, Tokyo, Japan) was carried out at 243 nm. Drug content was expressed as the amount of drug in unit weight of the microparticles.

### 2.12. Particle Size Analysis of Spray-Congealed Microparticles

Particle size measurement was carried out using an optical microscope (BX61, Olympus, Tokyo, Japan). Image analysis software (Image-Pro 6.3, Media Cybernetics, Rockville, MD, USA) was used to analyze 625 particles for each sample. The D_10_, D_50_, and D_90_, corresponding to the 10th, 50th, and 90th volume percentiles under the cumulative undersize distribution plot, were obtained. The span value represents the spread of particle size distribution and was obtained as follows:

Span = (D_90_ − D_10_)/D_50_. (3)

A higher span value indicates a broader size distribution.

### 2.13. Examination of Surface Solid-State Properties

Raman spectroscopy was used to elucidate the surface solid-state properties and degree of surface drug coating. The individual components and microparticles were examined using a Raman spectrometer (XploRA, Horiba Scientific, Longjumeau, France) equipped with a 532 nm He/Ne laser source (30 mW), 1200/1 nm grating and a microscope system (BX51, Olympus, Tokyo, Japan). Spectra collection was performed at room temperature under the following conditions: 10× microscope objective, 300 µm pinhole size, 100 µm slit width, and 1 s exposure time. Measurements were taken at a single spot on the center of the particle.

### 2.14. Drug Release Study

The drug dissolution profile of 1 g of the sample in phosphate buffer (pH 7.4) was determined with a dissolution test unit (9ST, Caleva, Sturminster Newton, UK) using the USP Apparatus 1. The basket rotation speed was set at 100 rpm. The volume of the dissolution medium used was 900 mL, and it was maintained at 37 °C Aliquots were withdrawn at specific time points and assayed for paracetamol using UV spectrophotometry. The dissolution was also carried out in the presence of a low concentration of surfactant to investigate its effect on drug release. Sodium lauryl sulphate was added into the dissolution medium to reduce the agglomeration of spray-congealed microparticles.

### 2.15. Statistical Analyses of Data

The results were analyzed at 5% level of significance using a statistical analysis software (SPSS Statistics 23.0, IBM, Armonk, NY, USA). The Wilcoxon signed-rank test was used to compare the observed and weighted viscosity of blends. The Wilcoxon rank-sum test was used to compare the melting points of the wax-lipid matrices before and after addition of paracetamol. The Kruskal-Wallis and Wilcoxon rank-sum tests were used to compare the particle sizes of spray-congealed microparticles.

## 3. Results and Discussion

### 3.1. Rheological Tests

The objective of the rheological tests was to compare the observed viscosity, henceforth referred to as “viscosity,” of wax-lipid blends with their weighted viscosity to determine the effect of lipid additives on viscosity. The weighted viscosity assumes that there are no interactions between the blend components, and is calculated according to the equation

(4) $$\begin{array}{c} \mathrm{Viscosity}\;\mathrm{of}\;\mathrm{blend}=\mathrm{Proportion}\;\mathrm{of}\;\mathrm{component}\;1\times \mathrm{Viscosity}\;\mathrm{of}\;\mathrm{component}\;1+\\ \mathrm{Proportion}\;\mathrm{of}\;\mathrm{component}\;2\times \mathrm{Viscosity}\;\mathrm{of}\;\mathrm{component}\;2 \end{array}$$

The rheograms of the individual materials and blends showed a straight line passing through the origin. This indicates a linear relationship between shear rate and shear stress, which is typical of Newtonian flow. The viscosity at 95 °C increased in the following order: CA < PW59 < CE < SA < PW84. The same trend was observed at 69 °C, excluding PW84.

The temperature ramping curves were biphasic in nature. As temperature increased initially, the viscosity decreased rapidly in an almost linear fashion. Beyond a certain temperature, the decrease in viscosity was still linear but with a gentler gradient. Similar observations were made in another study on various lipid-based materials [58]. It was explained that the alkyl chains were more easily aligned in the direction of shear as the molecules gained kinetic energy, and intermolecular attractions were weakened. Once the alkyl chains were aligned, further increase in temperature would have less effect on viscosity.

**Figure 2:**
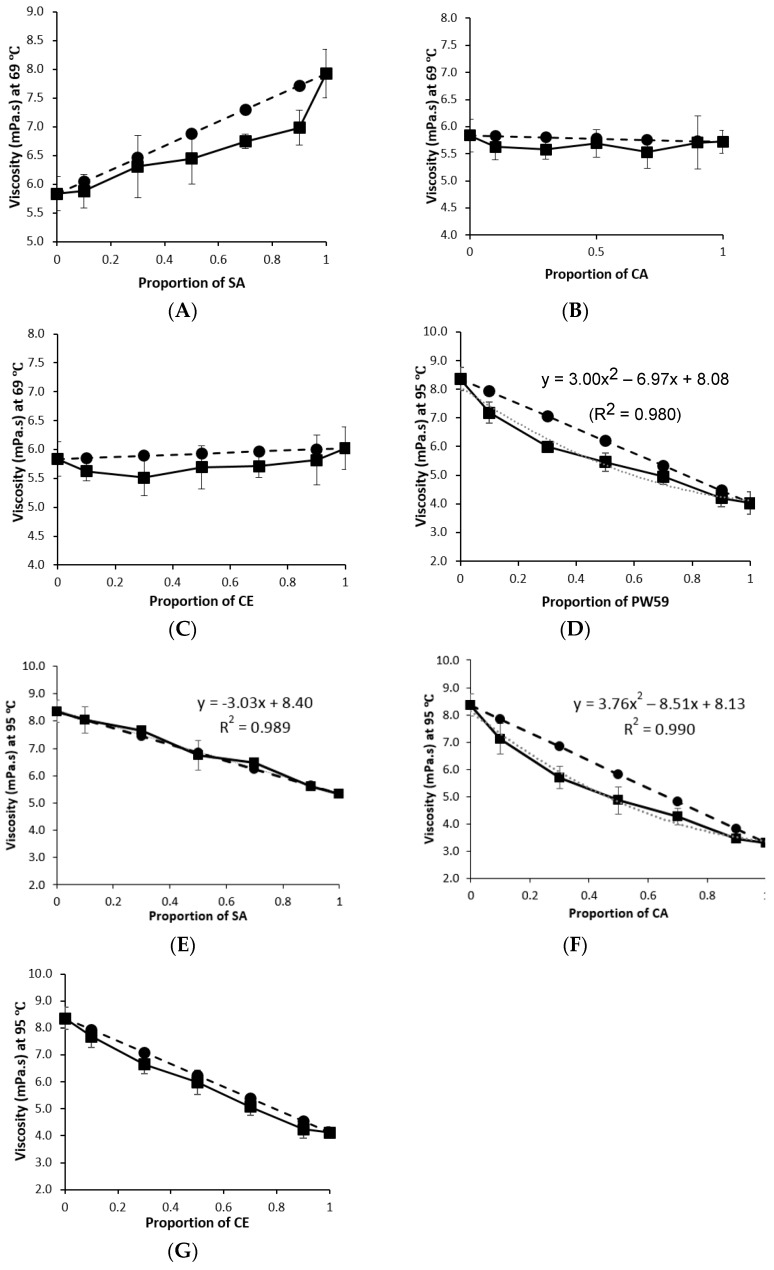
Plots of viscosity (■, solid line) and weighted viscosity (●, dashed line) against proportion of lipid additive in the (**A**) PW59:SA; (**B**) PW59:CA; (**C**) PW59:CE; (**D**) PW84:PW59; (**E**) PW84:SA; (**F**) PW84:CA; (**G**) PW84:CE blends (*n* = 3).

For most of the blends, their viscosity was in between those of the individual materials, and varied with the proportions of the individual materials present. However, the change in viscosity was not directly proportional to the change in proportion of the individual materials, suggesting interactions between the blend components. The blend viscosity was plotted against the proportion of lipid additive incorporated with paraffin wax, and the corresponding weighted viscosity is shown for comparison (Figure 2A–G). With the exception of the blends of PW84:SA, most of the viscosity was lower than their weighted viscosity, and analysis with the Wilcoxon signed-rank test showed that the difference was statistically significant (*p* = 0.043). The difference was not statistically significant for the blends of PW84:SA (*p* = 0.686), indicating that there was no significant interaction for PW84:SA blends.

### 3.2. FTIR Spectroscopy

The FTIR spectra of individual materials and blends are mainly presented in the Supplementary Materials (Figure S1). Although the FTIR spectroscopy was performed on samples in a solid state, similar interactions are expected to be present in the molten form, affecting the viscosity. All samples exhibited peaks in the 2960–2840 cm^−1^ range that corresponds to C–H stretch of long carbon chains. There were also peaks around 1400 cm^−1^ that corresponds to C–H deformation.

In addition, the SA spectrum showed a very broad and strong peak in the 3320–2500 cm^−1^ range, superimposed on the C–H stretch, which is in line with SA existing as hydrogen-bonded dimers [59]. Furthermore, there were O–H deformation peaks at 1430 and 1411 cm^−1^, a C=O stretch peak at 1698 cm^−1^, and C–O stretch peaks at 1310 and 1295 cm^−1^. The CA spectrum showed O–H stretch peaks at 3324 and 3225 cm^−1^, and C–O stretch peaks in the 1250–1000 cm^−1^ range. The CE spectrum exhibited a C=O stretch peak at 1732 cm^−1^, and C–O stretch peaks in the 1320–1000 cm^−1^ range.

**Figure 3:**
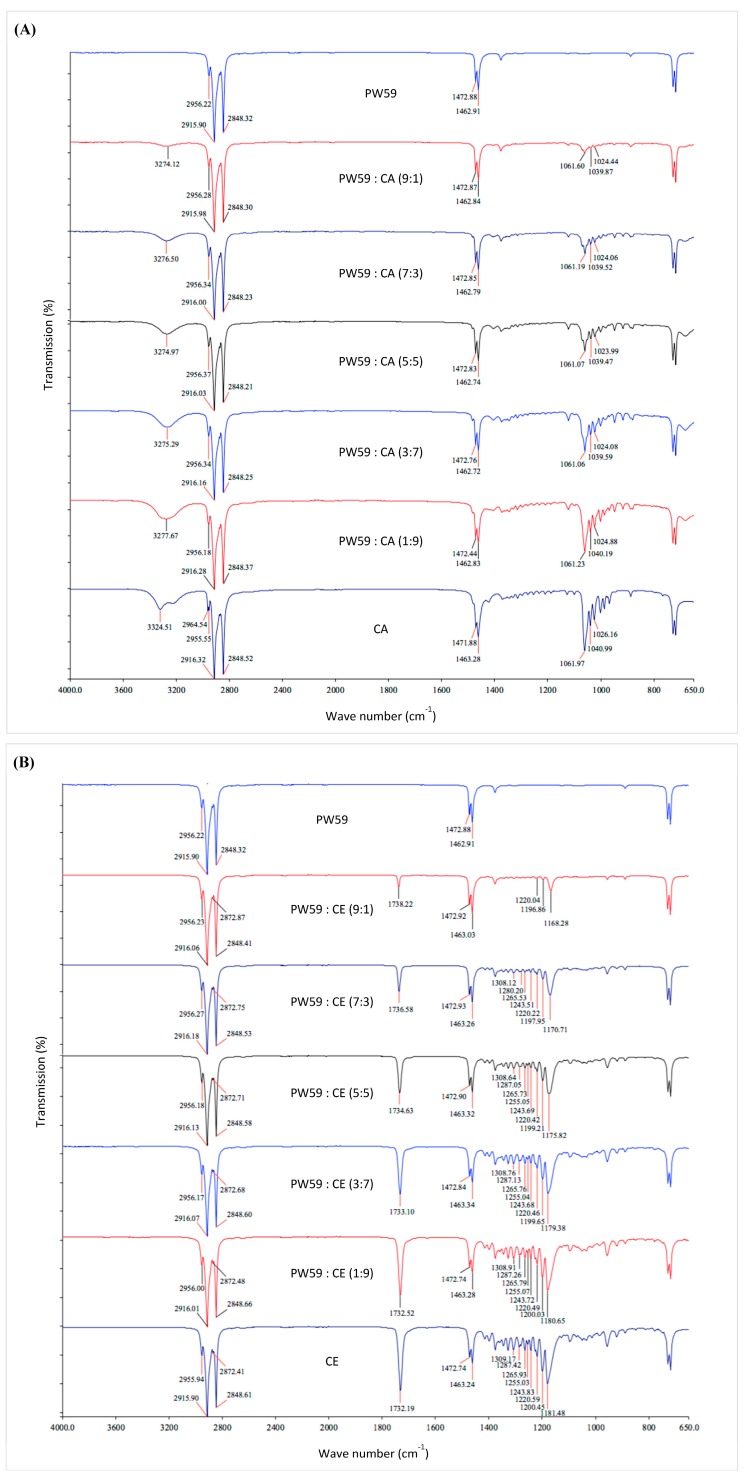
FTIR spectra of (**A**) PW59, CA, and blends; (**B**) PW59, CE, and blends (*n* = 3).

The spectra of blends were largely similar to the combinations of the spectra of the individual components in various proportions. There were only observable differences for the PW59:CA and PW59:CE blends (Figure 3).

For PW59:CA blends, the O–H peak of CA melded into a single broad band centered around 3275 cm^−1^ after the addition of PW59, compared to the original two peaks at 3324 and 3225 cm^−1^ (Figure 3A). This observation suggests that the hydroxyl groups of CA were no longer free. Due to the absence of hydroxyl groups in PW59 molecules, hydrogen bonding between PW59 and CA molecules would not be possible. Based on the molecular structures, permanent dipole-induced dipole bonding would be the most likely interactions.

For PW59:CE blends, there was a gradual shift in the C–O peak from 1181 to 1168 cm^−1^ when PW59 was present in increasing proportions. There was also a gradual shift in the C=O peak from 1732 to 1738 cm^−1^ (Figure 3B). These shifts indicate an interaction between PW59 and CE molecules, likely permanent dipole-induced dipole interactions.

There were no significant changes in the FTIR spectra for the remaining blends. However, this does not exclude the possibility of interactions between the materials when blended.

### 3.3. Conformations of Molecules under Shear

**Figure 4:**
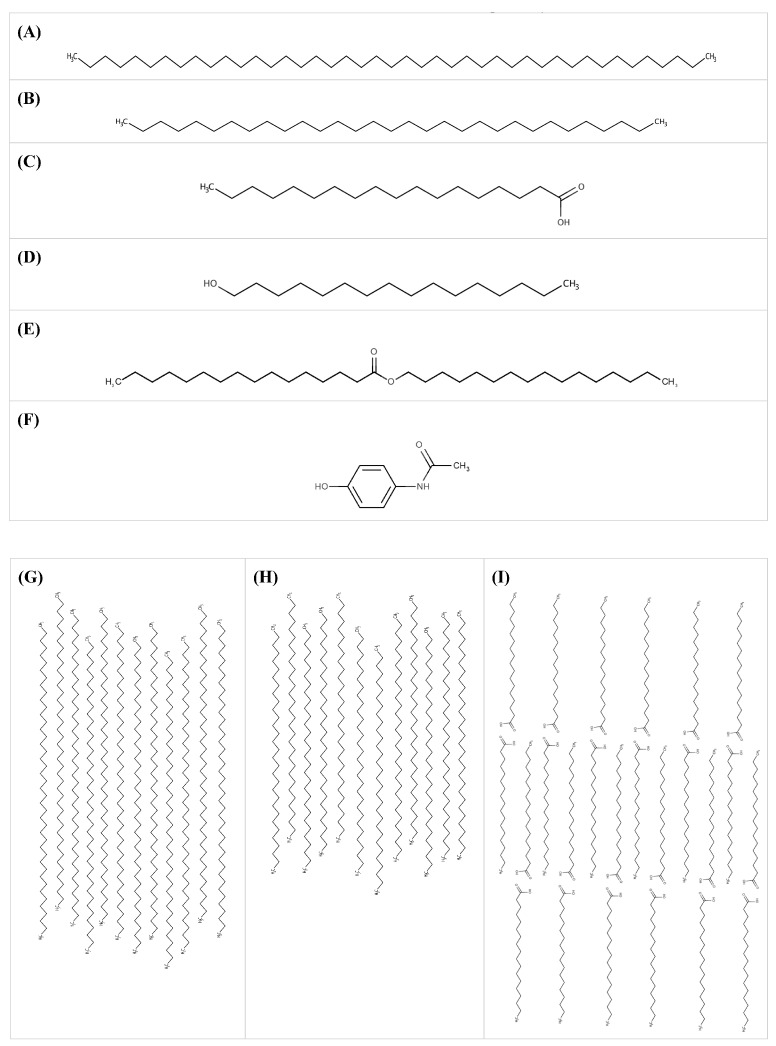

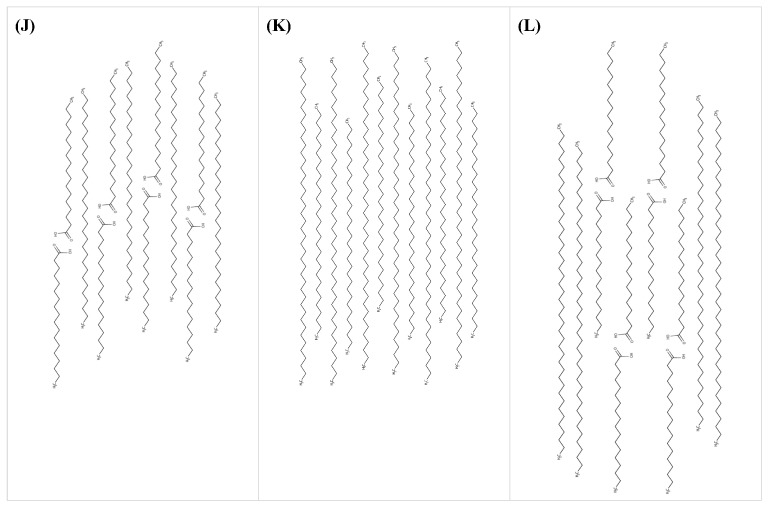
Molecular structures of (**A**) PW84; (**B**) PW59; (**C**) SA; (**D**) CA; (**E**) CE; (**F**) paracetamol; and representative postulated conformations of (**G**) PW84 molecules; (**H**) PW59 molecules; (**I**) SA molecules; (**J**) PW59:SA blend; (**K**) PW84:PW59 blend; and (**L**) PW84:SA blend under shear, drawn using MarvinSketch program (MarvinSketch 17.1.23.0, ChemAxon, Budapest, Hungary), which takes into consideration the energy of the conformation of molecules. Note: Only representative conformations are shown. The rest are presented in Supplementary Materials (Figure S2).

Studies have proposed a cluster model for SA homodimers in liquid state to allow for their compact packing [60] (Figure 4H). The other materials did not have studies reporting on their conformations in liquid state. Based on the experimental results from the rheological tests and FTIR studies, the molecules were most likely aligned under shear and not entangled as Newtonian flow was observed (Figure 4).

With the exception of PW84:SA blends, other blends had viscosity lower than the weighted viscosity. The differences could be attributed to the interspersing of paraffin wax molecules between the lipid, resulting in the disruption of cohesive forces and replacement with weaker adhesive forces. Amongst these blends, PW59:CA and PW59:CE had observable changes in their FTIR spectra. The newly formed permanent dipole-induced dipole interactions between PW59 and CA or CE molecules were weaker than the original dipole-dipole interactions between CA or CE molecules. The remaining blends did not exhibit any obvious changes in their FTIR spectra. For PW59:SA blends, PW59 molecules disrupted the cohesive interactions between the SA dimers. When PW59 was blended with PW84, the former would intersperse between the PW84 molecules, disrupting the extent of van der Waals forces between PW84 molecules. This trend also applied to PW84:CA, as well as PW84:CE, where the shorter chains of CA or CE molecules would intersperse and disrupt the van der Waals forces between PW84 molecules.

PW84:SA blends showed no statistically significant difference between the viscosity and the weighted viscosity, and no observable changes in the FTIR spectra. As PW84 is a long hydrocarbon chain and SA exists as dimers, it was likely more difficult for them to intersperse and disrupt the cohesive interactions between PW84 molecules and SA dimers, respectively.

### 3.4. Modeling the Viscosity of Blends

For PW84:SA blends, the viscosity was found to vary with the proportions of the individual materials present, according to a linear relationship: *y* = –3.03*x* + 8.40 (*R*^2^ = 0.989), where y represents the blend viscosity and *x* represents the proportion of lipid additive present (Figure 2E). There was no significant difference between viscosity and weighted viscosity as there was no interaction, hence the blend viscosity could also be predicted based on weighted viscosity.

For the rest of the blends, the relationship between the viscosity and proportion of lipid additive was less straightforward. For example, the PW84:PW59 as well as the PW84:CA blends were best represented by quadratic formulae: *y* = 3.00*x*^2^ – 6.97*x* + 8.08 (*R*^2^ = 0.980) and *y* = 3.76*x*^2^ – 8.51*x* + 8.13 (*R*^2^ = 0.990) respectively (Figure 2D,F). The blend viscosity could not be predicted based on weighted viscosity due to interactions between the individual materials as the wax hydrocarbons lubricated the flow of the higher viscosity lipids. Hence, it was difficult to develop a universal model to predict the viscosity of blends.

### 3.5. Rheological Tests with Drug

**Figure 5:**
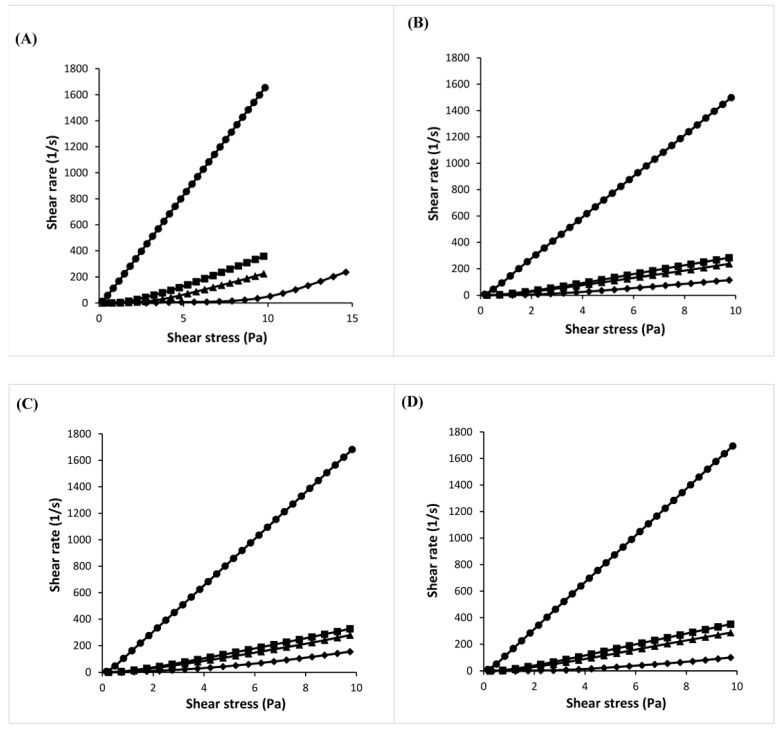
Rheograms of (**A**) PW59; (**B**) PW59:SA (1:1); (**C**) PW59:CA (1:1); (**D**) PW59:CE (1:1) systems containing 0% (●); 5% (■); 10% (▲); 20% *w*/*w* (♦) paracetamol (*n* = 3). Note: Although this is not clearly seen in the rheograms, the drug-loaded blends comprising PW59 and SA, CA, or CE exhibited pseudoplastic flow as the rheometer readings showed that the samples moved as soon as a stress was applied.

The effect of the lipid additives in lowering the viscosity of drug-loaded paraffin wax was investigated using paracetamol with the following particle size range: D_10_ = 5.0 µm, D_50_ = 20.2 µm, and D_90_ = 49.9 µm. The drug-loaded PW59 systems exhibited plastic flow (Figure 5A). Appreciable flow began only after a shear stress equivalent to the yield value was exerted, but at shear stress above yield value, the flow resembled a Newtonian system. The yield value increased from 1 Pa at 5% paracetamol, to 2 Pa at 10% paracetamol, and 4 Pa at 20% paracetamol. This indicates greater resistance to flow at higher drug load due to the formation of a stronger network of drug particles. However, the plastic flow observed at a low drug load of 5% was unexpected as the amount of drug particles was unlikely to be able to form a network. It was observed that paracetamol was difficult to disperse uniformly in PW59 as it was lyophobic and tended to aggregate. This could give rise to inaccuracies when conducting rheological tests.

Drug-loaded blends comprising PW59 and SA, CA, or CE exhibited pseudoplastic flow (Figure 5B–D). The rheograms were characterized by slightly concave curves, whereby viscosity decreased with increasing shear stress. As the shear stress continued to increase, the flow curve tended towards linearity. The viscosity increased with increasing drug load, as indicated by the gentler slopes of the rheograms (Figure 5). Their apparent viscosity was calculated from the tangent to the curve at three different shear stress using Matlab R2015b 8.6.0 (The Mathworks Inc., Natick, MA, USA) program (Table 1).

For formulations containing 20% drug load, PW59 had the highest viscosity at all three shear stress. When lipid additives were present, the viscosity was lowered significantly (Table 1), making the formulation more amenable for spray congealing by improving flow and reducing the chances of clogging.

An equation of flow applicable to colloidal dispersions of spherical particles was developed by Einstein as follows:

η = η_0_ (1 + 2.5φ) (5)

where η_0_ is the viscosity of the dispersion medium and η the viscosity of the dispersion when the volume fraction of colloidal particles present is φ. At the shear stress of 8 Pa, the viscosities of the four formulations at 20% drug concentration can be obtained and the respective volume fractions calculated. (Table 2) The volume fractions present decreased in the following order: PW59 > PW59:CE > PW59:SA > PW59:CA.

As volume fraction is directly related to concentration, a lower concentration indicated that more drug is partially solubilized in the matrix. The higher partial solubility of paracetamol in the PW59:SA and PW59:CA matrices resulted in lower volume fractions of drug particles present, hence lowering the viscosity to greater extents.

### 3.6. Thermal Analysis

**Figure 6:**
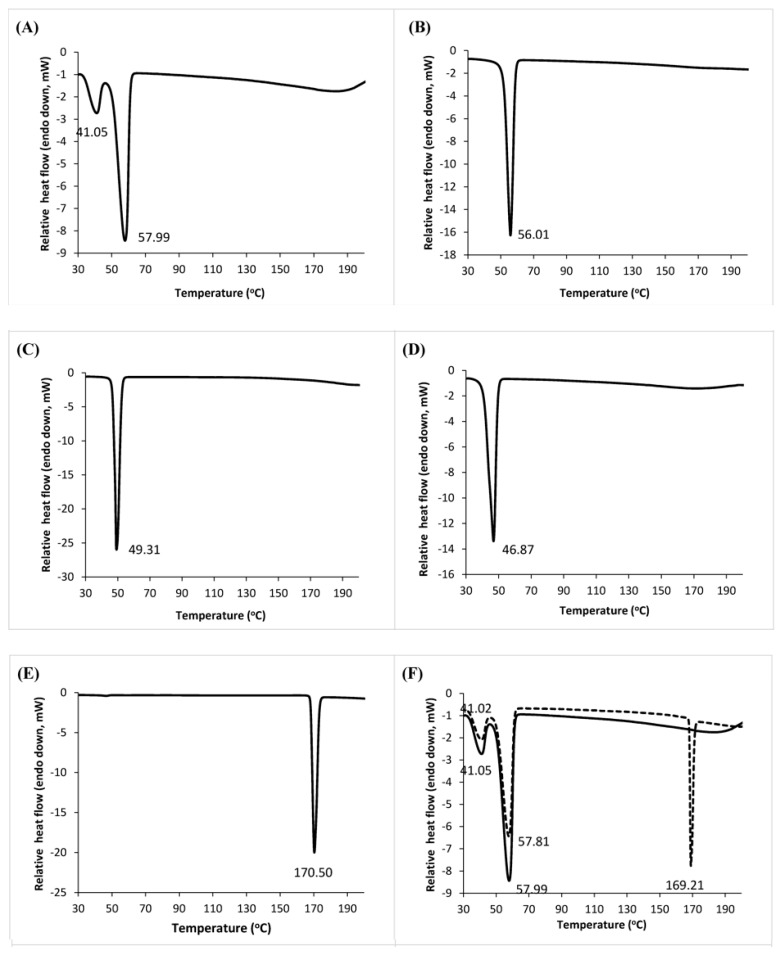

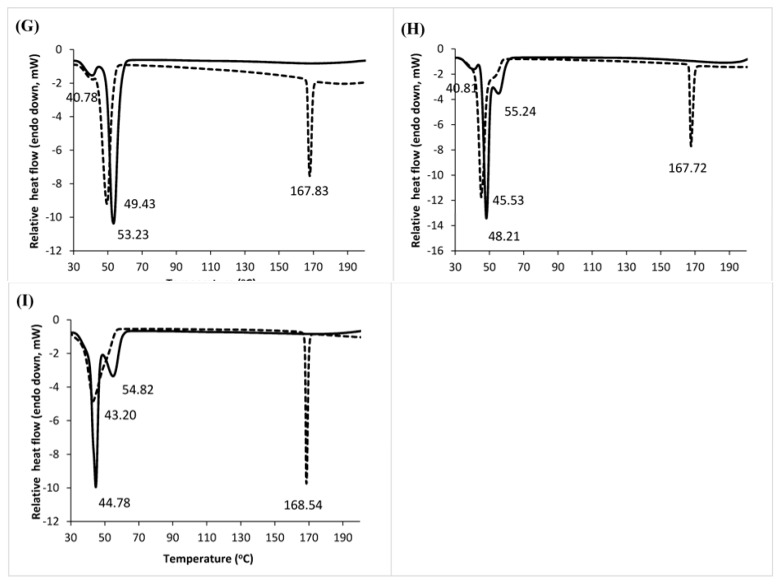
DSC thermograms of (**A**) PW59 unprocessed matrix; (**B**) SA unprocessed matrix; (**C**) CA unprocessed matrix; (**D**) CE unprocessed matrix; (**E**) paracetamol; (**F**) PW59; (**G**) PW59:SA (1:1); (**H**) PW59:CA (1:1); (**I**) PW59:CE (1:1). The dashed lines represent thermograms with 20% *w*/*w* paracetamol (*n* = 3).

Figure 6A–E shows the DSC curves of the single components. Two endotherms, which represent the melting points (41.05 and 57.99 °C), were present on the DSC curve of PW59. For SA, only one melting endotherm was observed at 56.01 °C. CA showed a strong endothermic peak at 49.31 °C, while CE showed a strong peak at 46.87 °C. For paracetamol, the DSC thermogram showed that it melted at 170.50 °C.

The melting point of a matrix is lowered by the presence of solute in a colligative way, where the extent of depression is proportional to the solute concentration. When the solute concentration exceeds its solubility limit in the matrix, the solute will exist as discrete particles or crystals. The extent of solubility of paracetamol within molten lipid will affect the melting point depression of the molten lipid caused by the partially solubilized paracetamol.

The melting endotherm of paracetamol was present in the DSC thermograms of all spray-congealed formulations with 20% *w*/*w* paracetamol. This suggests that paracetamol did not fully dissolve in the molten lipid matrices upon matrix melting, and there was no amorphization of paracetamol in the lipid matrices. At this concentration of paracetamol, its solubility in molten lipid had been exceeded. Undissolved particles of paracetamol remained and produced the characteristic drug melting peak. The drug is likely to be present as a dispersion of small crystalline particulates within the molten matrix.

Spray-congealed PW59 microparticles containing 20% *w*/*w* paracetamol showed a very slight, insignificant depression in the melting points of PW59 from 41.05 to 41.02 °C and 57.99 to 57.81 °C (*p* = 0.827) (Figure 6F). This may be explained by a partial dissolution of paracetamol in the melting matrix [61]. Compared to the corresponding matrices without drug, the melting point of CA was depressed significantly from 48.21 to 45.53 °C (*p* = 0.046) (Figure 6H). For SA, its melting point was depressed too, albeit to a non-significant extent, from 53.23 to 49.43 °C (*p* = 0.050) (Figure 6G). In contrast, the melting point of CE showed a slight, insignificant depression from 44.78 to 43.20 °C (*p* = 0.050) (Figure 6I). The greater depression in the melting points of CA and SA indicated that paracetamol had higher solubility in these two lipids compared to CE.

In addition, the two melting peaks of PW59, originally centred at 41.05 and 57.99 °C, both remained relatively unchanged when only paracetamol was added to it (Figure 6F). When SA, CA and CE were used as lipid additives, both peaks disappeared, which indicated that paracetamol had partial solubility in these molten matrices, which would have effect on depressing the melting points of the matrices (Figure 6G–I). This partial solubility of paracetamol could explain the reduction in viscosity when lipid additives were added to the drug-loaded PW59 matrix. The higher extent of solubility led to a greater reduction in viscosity when SA and CA were used as lipid additives compared to CE, which was supported by the lower volume fractions obtained from rheological tests.

### 3.7. Total and Useful Yields of Spray-Congealed Microparticles

Spray congealing was successfully used to produce microparticles from all four formulations. All formulations achieved total and useful yields greater than 80% and 74% respectively (Table 3). This was in concurrence with previous studies, which reported that spray congealing was able to attain high total yields of 90% or higher [1,17]. The loss in total yield was largely due to sticking of the microparticles onto the chamber wall while a lower useful yield was attributed to losses through the production of fines (<10 μm), which were collected separately in the cyclone. The use of lipid additives reduced the sticking of microparticles onto the chamber wall and resulted in higher total and useful yields compared to the formulation that had only PW59.

### 3.8. Surface Characteristics of Spray-Congealed Microparticles

**Figure 7:**
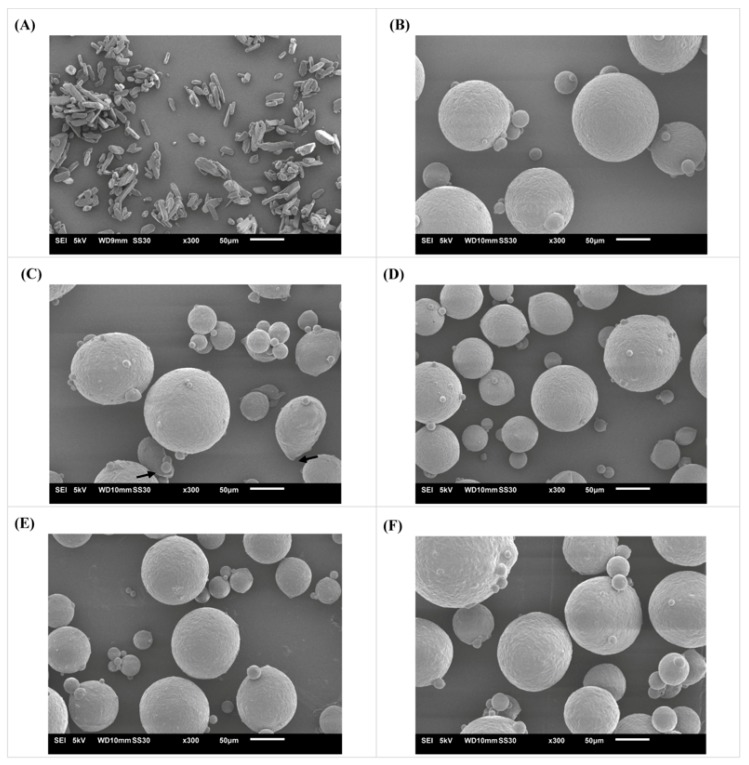
Scanning electron microscope images of (**A**) paracetamol; (**B**) blank PW59 microparticles; (**C**) 20% *w*/*w* paracetamol-loaded spray-congealed PW59 microparticle; (**D**) 20% *w*/*w* paracetamol-loaded spray-congealed PW59:SA microparticle; (**E**) 20% *w*/*w* paracetamol-loaded spray-congealed PW59:CA microparticle; (**F**) 20% *w*/*w* paracetamol-loaded spray-congealed PW59:CE microparticle.

Paracetamol existed as elongated, needle-like crystals of various sizes. For all formulations, spray-congealed microparticles in the useful fraction were generally discrete, dense and spherical in nature (Figure 7). Microparticles composed of only pure PW59 matrix (blank microparticles) were noted to be relatively smooth (Figure 7B). The addition of lipid additives and 20% *w*/*w* paracetamol did not affect the microparticle surface significantly and minimal protuberances were observed. However, a number of the drug-loaded spray-congealed PW59 microparticles had protruded drug particles (arrows in Figure 7C), which indicates that some paracetamol particles were not well encapsulated by paraffin wax. The addition of lipid additives allowed for better surface drug coating.

### 3.9. Drug Content

Although the theoretical drug content was 20%, all test samples were found to have drug contents of 21.6% to 21.9% (Table 3). This indicates that spray congealing of PW59 and blends can produce microparticles with satisfactory drug content. However, the drug content was higher than the theoretical amount. Previous studies also obtained similar findings [33,37,48] and a recent study attributed it to non-uniformity in drug concentration within the microparticles [62]. Very small microparticles were formed only by the matrix (empty spheres) that did not contain the drug, and these were collected as fines in the cyclone. In the present work, the loss of PW59 and lipid additives to the delivery tube might have resulted in a higher drug concentration when the formulation was atomized. Another possible reason could be the loss of larger microparticles during spray congealing, as they would take a longer time to solidify and adhere to the chamber wall upon impact. These larger microparticles were likely to contain more PW59 and lipid additives compared to the drug, hence the yield collected would consist of relatively smaller microparticles with higher drug content.

### 3.10. Particle Size Characteristics

Particle size distributions were found to be non-parametric in nature, hence the D_10_, D_50_, D_90_, and span values are reported (Table 4). The Kruskal-Wallis test was performed to investigate the influence of various formulations on the particle size of the spray-congealed microparticles, and the results showed a statistically significant difference in particle size (*p* = 0.000) (Table 4). Pairwise comparisons were conducted using the Wilcoxon rank-sum test to determine which pairs were significantly different. The significance level for each pairwise comparison after Bonferroni adjustment was $\frac{0.05}{6}$ = 0.00833. 

The difference in particle size was significant between PW59 without lipid additive and PW59:CA (*p* = 0.000). The difference was close to significant between PW59 without lipid additive and PW59:SA (*p* = 0.016), and between PW59 without lipid additive and PW59:CE (*p* = 0.014). In contrast, the differences between the other pairs were insignificant (Table 4). It could be inferred that the lipid additives resulted in smaller spray-congealed microparticles, due to the lowering of viscosity. In addition, the size span decreased as the size of microparticles decreased. The use of lipid additives may result in a smaller range of microparticle sizes, allowing more consistent drug release.

### 3.11. Surface Solid-State Properties

**Figure 8:**
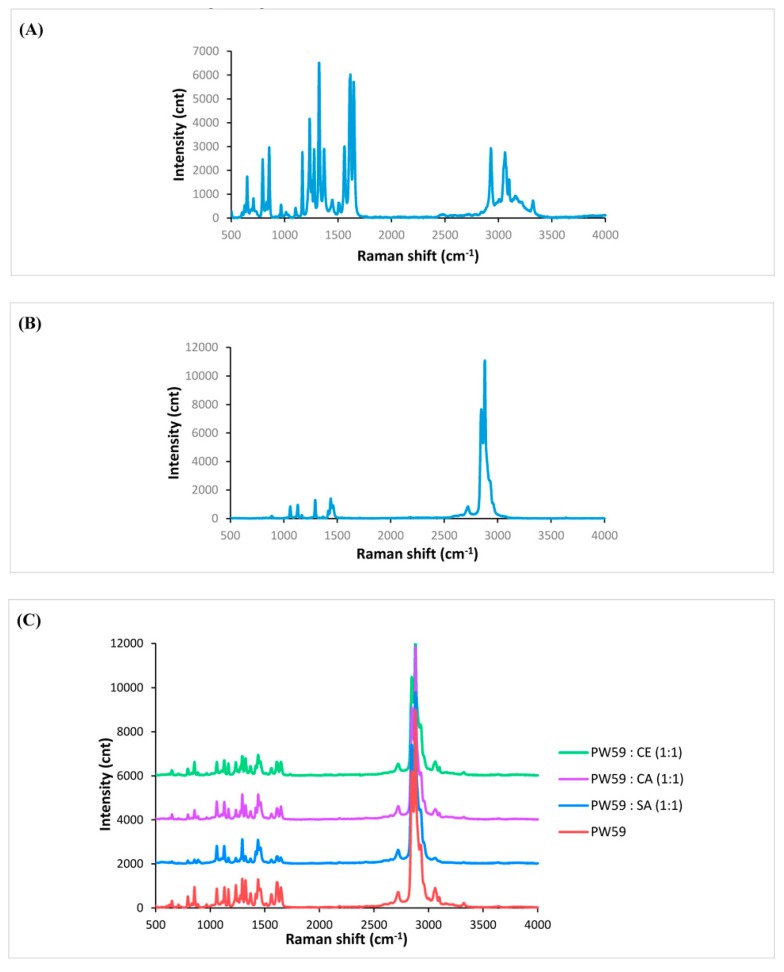
Raman spectra of (**A**) paracetamol, (**B**) PW59, and (**C**) spray-congealed microparticles (*n* = 3). Note: The Raman spectra of SA, CA, and CE were similar to PW59, so only the PW59 spectrum is shown.

Raman spectroscopy was used to investigate the degree of surface drug coating in the microparticles. Both PW59 and paracetamol displayed characteristic Raman spectra (Figure 8A,B). Most of the peaks in the Raman spectra of PW59 and the lipid additives correspond to mainly alkyl groups [63]. Hence, the polar functional groups of the lipid additives do not play a significant role in the Raman spectra. As a result, the Raman spectra of CA, SA, and CE were similar to that of PW59, so only the PW59 spectrum is shown. The Raman spectra of paracetamol exhibited characteristic peaks around 1610 and 1646 cm^−1^. 

PW59 without lipid additive showed the most intense drug peaks, indicating that drug particles were exposed and not well coated (Figure 8C). In comparison, PW59 with lipid additives showed less intense drug peaks. Thus, it is likely that the drug was better coated with the use of lipid additives. In particular, PW59:SA and PW59:CA had drug peaks of the lowest intensity. It should be recalled that the lipid additives have polar functional groups, which could form hydrogen bonds with the drug particles and accounted for the better surface drug coating. SA and CA being more polar than CE, resulted in better drug coating.

### 3.12. Drug Release Study

**Figure 9:**
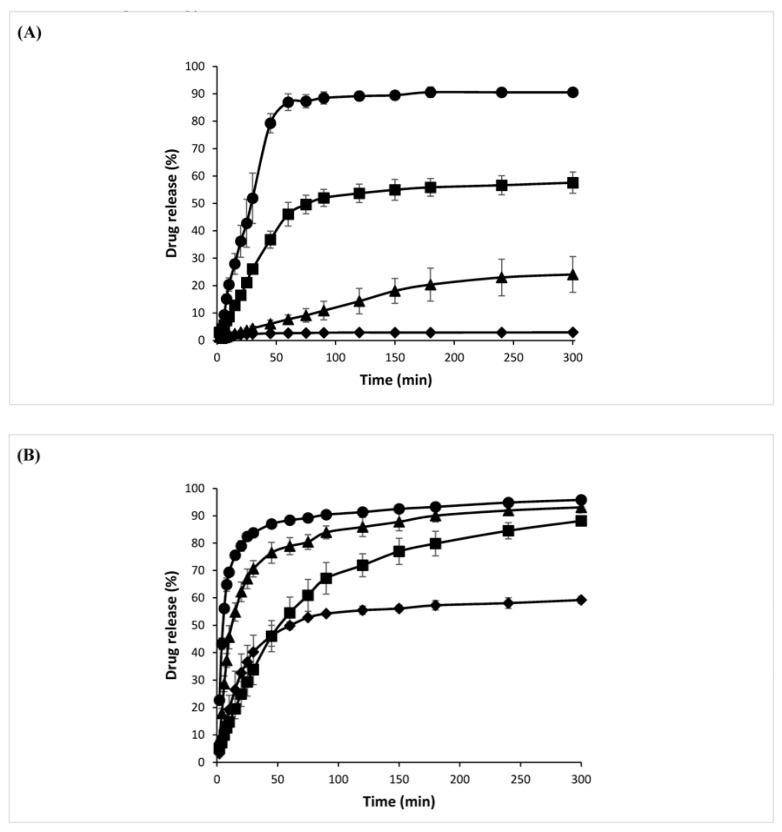
Drug release profiles of spray-congealed microparticles containing 20% *w*/*w* paracetamol for PW59:SA (●); PW59:CA (■); PW59:CE (▲); PW59 (♦) in the (**A**) absence and (**B**) presence of 0.05% *w*/*v* sodium lauryl sulphate (*n* = 3).

All four formulations sustained drug release sufficiently such that the cumulative drug release was lower than 5% up to the 4-min mark, showing suitability of PW59 and blends for taste-masking. PW59 without lipid additive and PW59:CE produced microparticles with slow drug release, and the cumulative drug release was poor even after 5 h (2.9% and 24.1% respectively, Figure 9A). In contrast, PW59:SA and PW59:CA produced microparticles with faster and greater cumulative drug release (90.5% and 57.6% respectively), hence these formulations are more suitable for taste-masking while ensuring adequate drug release.

The microparticles produced from PW59 without lipid additive showed very slow and low drug release. Based on aqueous solubility data, the order of hydrophobicity of the lipid additives is as follows: CE > CA > SA. The addition of less hydrophobic lipid additives enhanced drug release markedly. The formation of pores due to the dissolution of the drug or lipid additives on the surface facilitated the release of drug from the interior of the microparticles. In particular, the fast erodibility of stearic acid [37] resulted in PW59:SA having the highest rate and extent of drug release among all the formulations.

In the presence of 0.05% *w*/*v* SLS, the drug release reached 95.8% for PW59:SA, compared to only 90.5% without the surfactant (Figure 9B). The drug release for the other three formulations also increased greatly, as the surfactant enabled wetting of the microparticles and reduced their clumping in the dissolution medium. This could allow a more representative comparison of the drug release of the different formulations without being affected by clumping. However, due to the increased wetting by the surfactant, 22.7% of the cumulative drug release was reached by the 2-min mark for PW59:SA. Interestingly, the drug release for PW59:CA was slower throughout as compared to PW59:CE. In fact, the drug release for PW59:CA was even slower than PW59 from the 6th minute to 45th minute. This could be due to better coating of the paracetamol drug particles by PW59:CA, and thus less drug was exposed on the surface of the microparticles, which was supported by results from Raman spectroscopy.

## 4. Conclusions

The viscosity of wax-lipid blends was found to differ from the weighted viscosity when interactions occurred between the constituents in the blends. This difference could be explained using FTIR spectroscopy and postulated conformations of the molecules under shear. The relationship between viscosity and the proportion of lipid additive present was not straightforward, and it would be difficult to develop a universal model to predict the blend viscosity. Nonetheless, lipid additives can lower the viscosity of blends with paraffin wax, enabling efficient and successful spray congealing. The addition of paracetamol (20%) to molten paraffin wax changed the rheological property from Newtonian to plastic flow. When lipid additives (40%) were added to the drug-loaded paraffin wax, the blends exhibited pseudoplastic flow with lower viscosity and were more amenable for spray congealing. Compared to only paraffin wax, the addition of lipid additives resulted in smaller microparticles. Drug content remained high. Drug release and degree of drug coating were also higher due to lower hydrophobicity of the resultant matrix, which enabled better coating of the hydrophilic drug particles. The different lipid additives showed varying impact. Therefore, paraffin wax and blends are suitable matrix materials for the development of drug delivery systems by spray congealing. The lipid additives altered the rheological properties and hydrophobicity of paraffin wax and are useful for modifying the microparticle properties.

## Figures and Tables

**Table 1:** Viscosity at different shear stress for formulations containing various drug loads (*n* = 3).

	**Viscosity at Different Shear Stress (mPa.s) for 5% Drug Load**		
	**Shear Stress = 2 Pa**	**Shear Stress = 5 Pa**	**Shear Stress = 8 Pa**
PW59	36.6	22.9	20.3
PW59:SA (1:1)	37.2	29.6	30.7
PW59:CA (1:1)	33.0	27.4	25.3
PW59:CE (1:1)	29.5	24.1	24.7
	**Viscosity at Different Shear Stress (mPa.s) for 10% Drug Load**		
	**Shear Stress = 2 Pa**	**Shear Stress = 5 Pa**	**Shear Stress = 8 Pa**
PW59	165.3	32.9	29.7
PW59:SA (1:1)	44.4	36.6	35.6
PW59:CA (1:1)	40.0	31.9	28.6
PW59:CE (1:1)	38.3	28.6	29.8
	**Viscosity at Different Shear Stress (mPa.s) for 20% Drug Load**		
	**Shear Stress = 2 Pa**	**Shear Stress = 5 Pa**	**Shear Stress = 8 Pa**
PW59	1238.9	851.9	116.9
PW59:SA (1:1)	129.9	68.0	62.2
PW59:CA (1:1)	122.4	58.8	40.6
PW59:CE (1:1)	301.4	81.2	76.3

**Table 2:** Viscosities and volume fractions of the 20% drug-loaded formulations at shear stress of 8 Pa.

Formulation	η_0_ (mPa.s)	η (mPa.s)	φ
PW59	5.836	116.9	7.61
PW59:SA (1:1)	6.446	62.2	3.46
PW59:CA (1:1)	5.691	40.6	2.45
PW59:CE (1:1)	5.692	76.3	4.96

**Table 3:** Yields and drug content of microparticles produced from different drug-loaded formulations spray-congealed under similar conditions (0.3 bar pressure, 25% airflow, atomizing air temperature 10 °C above peak melting temperature of material, *n* = 3).

Formulation	Total Yield (%)	Useful Yield (%)	Fines (%)	Drug Content (%)
PW59	80.7 ± 4.8	74.5 ± 5.7	6.2 ± 0.9	21.8 ± 0.7
PW59:SA (1:1)	94.1 ± 2.1	87.5 ± 2.3	6.7 ± 0.3	21.6 ± 0.1
PW59:CA (1:1)	89.6 ± 4.5	81.1 ± 5.0	8.5 ± 0.6	21.6 ± 0.1
PW59:CE (1:1)	86.4 ± 0.3	81.5 ± 0.7	4.9 ± 0.5	21.9 ± 0.8

± standard deviation.

**Table 4:** Results of microparticle size characteristics (*n* = 3), Kruskal-Wallis test, and Wilcoxon rank-sum test.

Microparticle Size Characteristics				
	D_10_ (µm)	D_50_ (µm)	D_90_ (µm)	Span
PW59	23.05 ± 1.54	46.81 ± 2.21	106.27 ± 8.49	1.79 ± 0.30
PW59:SA (1:1)	20.93 ± 1.94	44.33 ± 0.95	92.57 ± 5.98	1.62 ± 0.17
PW59:CA (1:1)	19.51 ± 1.28	42.25 ± 3.76	85.34 ± 10.84	1.55 ± 0.14
PW59:CE (1:1)	20.20 ± 0.54	45.57 ± 2.52	93.06 ± 8.07	1.60 ± 0.09
**Test Groups**		**Kruskal-Wallis *p*-Value**		
PW59, PW59:SA (1:1), PW59:CA (1:1), PW59:CE (1:1)		0.000		
**Test Groups**		**Wilcoxon Rank-Sum *p*-Value**		
PW59, PW59:SA (1:1)		0.016		
PW59, PW59:CA (1:1)		0.000		
PW59, PW59:CE (1:1)		0.014		
PW59:SA (1:1), PW59:CA (1:1)		0.058		
PW59:SA (1:1), PW59:CE (1:1)		0.997		
PW59:CA (1:1), PW59:CE (1:1)		0.056		

± standard deviation.

## Supplementary Materials

The following are available online at http://www.mdpi.com/1999-4923/10/3/75/s1. Figure S1: FTIR spectra of (A) PW59, SA and blends; (B) PW84, PW59 and blends; (C) PW84, SA and blends; (D) PW84, CA and blends; (E) PW84, CE and blends (*n* = 3), Figure S2: Postulated conformations of (A) CA molecules; (B) CE molecules; (C) PW59 and CA blend; (D) PW59 and CE blend; (E) PW84 and CA blend; (F) PW84 and CE blend under shear, drawn using MarvinSketch program (MarvinSketch 17.1.23.0, ChemAxon, Hungary), which takes into consideration the energy of the conformation of molecules.
