# Supplementary material for: Effect of Lipid Additives and Drug on the Rheological Properties of Molten Paraffin Wax, Degree of Surface Drug Coating, and Drug Release in Spray-Congealed Microparticles
Source: Pharmaceutics. 2018 Jun 26;10(3):75. doi: 10.3390/pharmaceutics10030075 (PMC6160941; doi:10.3390/pharmaceutics10030075)
Supplement: Supplementary file 1 [file pharmaceutics-10-00075-s001.pdf]

pharmaceutics

Article

Effect of Lipid Additives and Drug on the Rheological Properties of Molten Paraffin Wax, Degree of Surface Drug Coating and Drug Release in Spray-Congealed Microparticles

Hongyi Ouyang, Audrey Yi Zheng, Paul Wan Sia Heng and Lai Wah Chan *

(A)

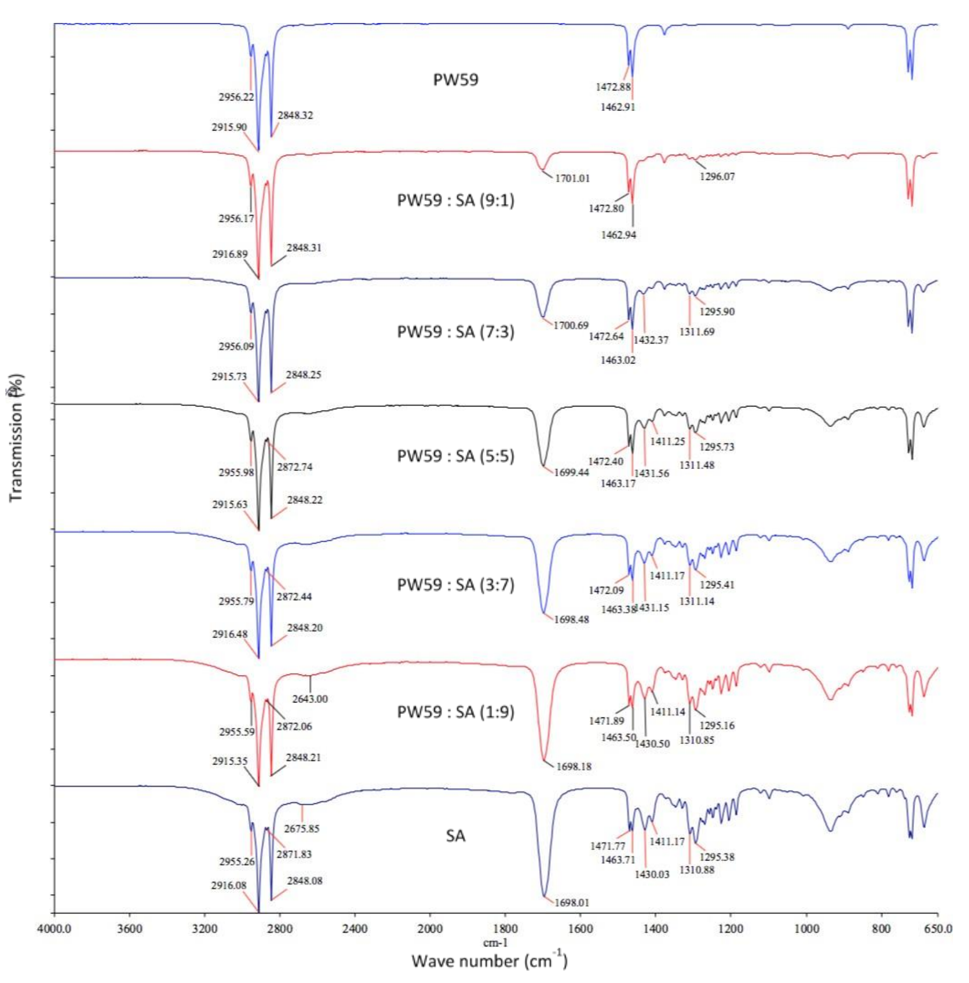

Pharmaceutics **2018**, *10*, 75; doi:10.3390/pharmaceutics10030075 www.mdpi.com/journal/pharmaceutics

Figure S1. *Cont.*

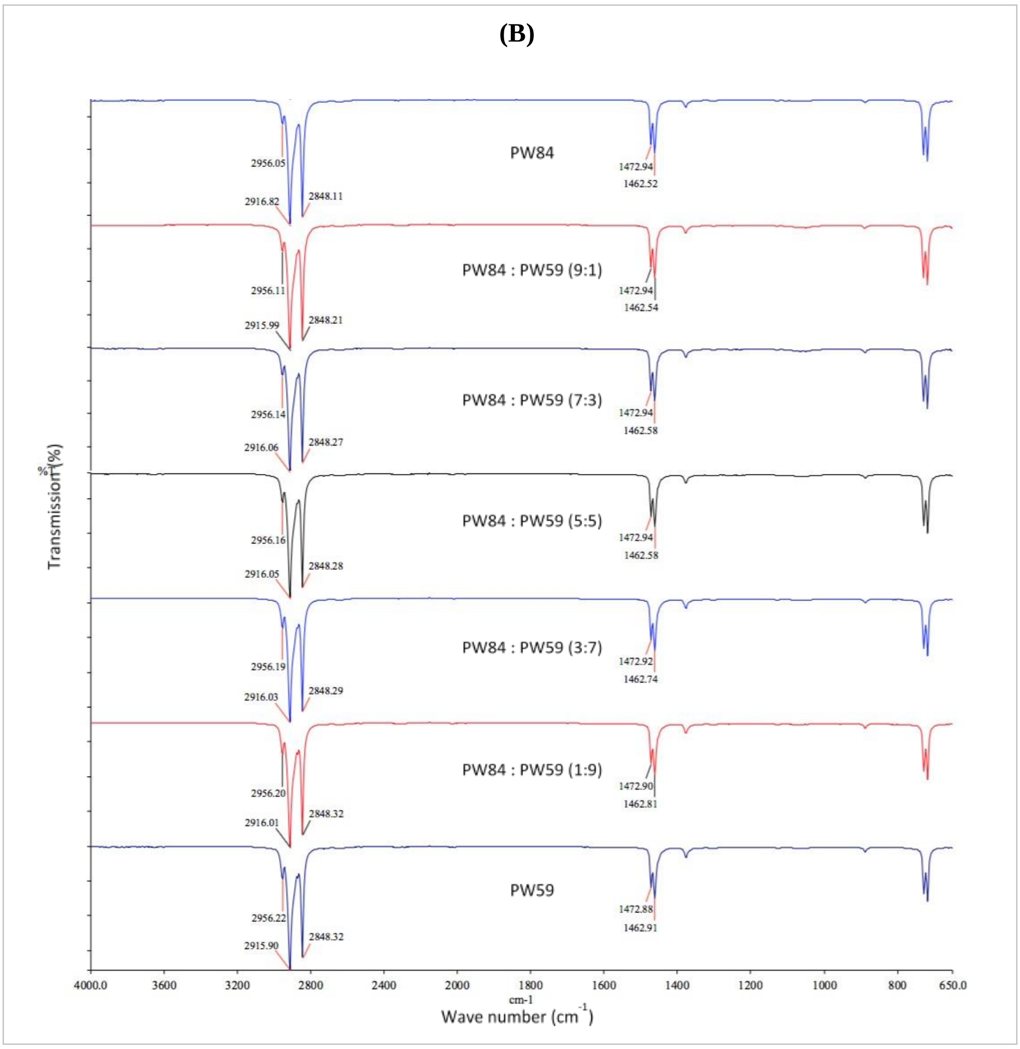

Figure S1. *Cont.*

(C)

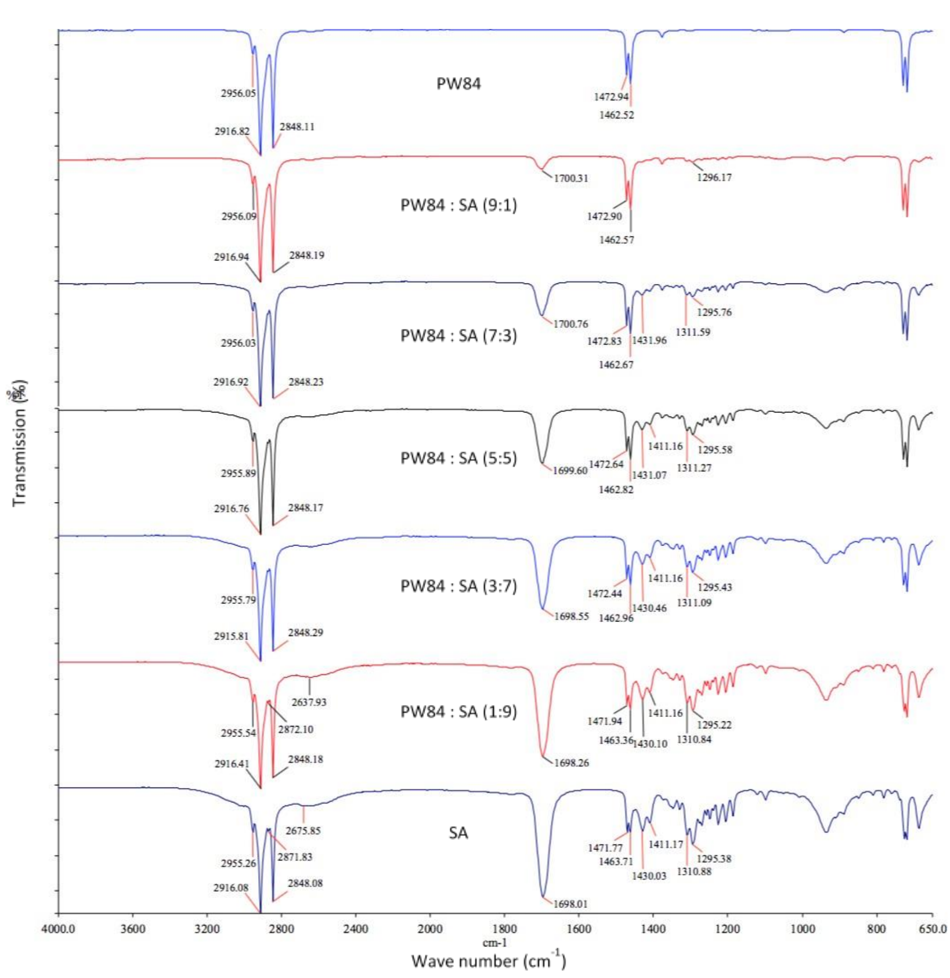

Figure S1. *Cont.*

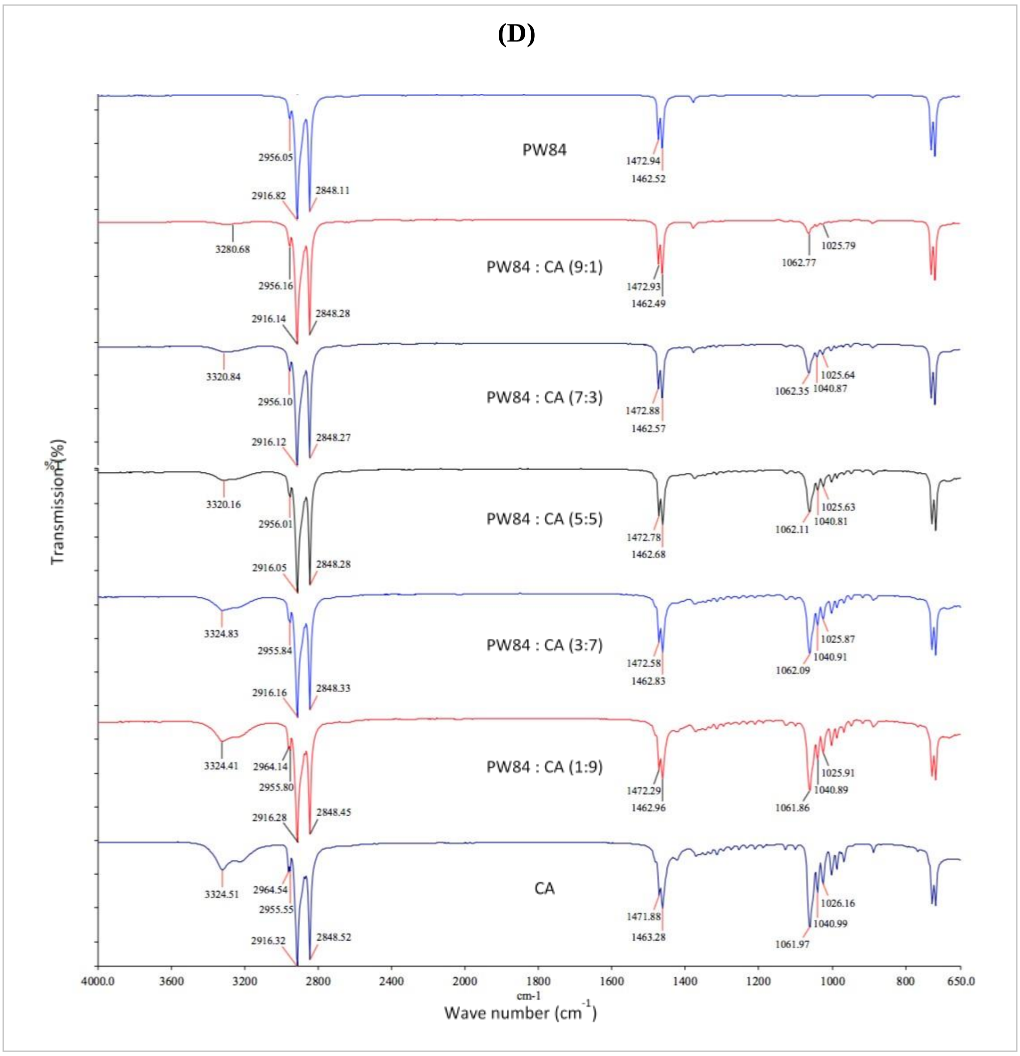

Figure S1. *Cont.*

(E)

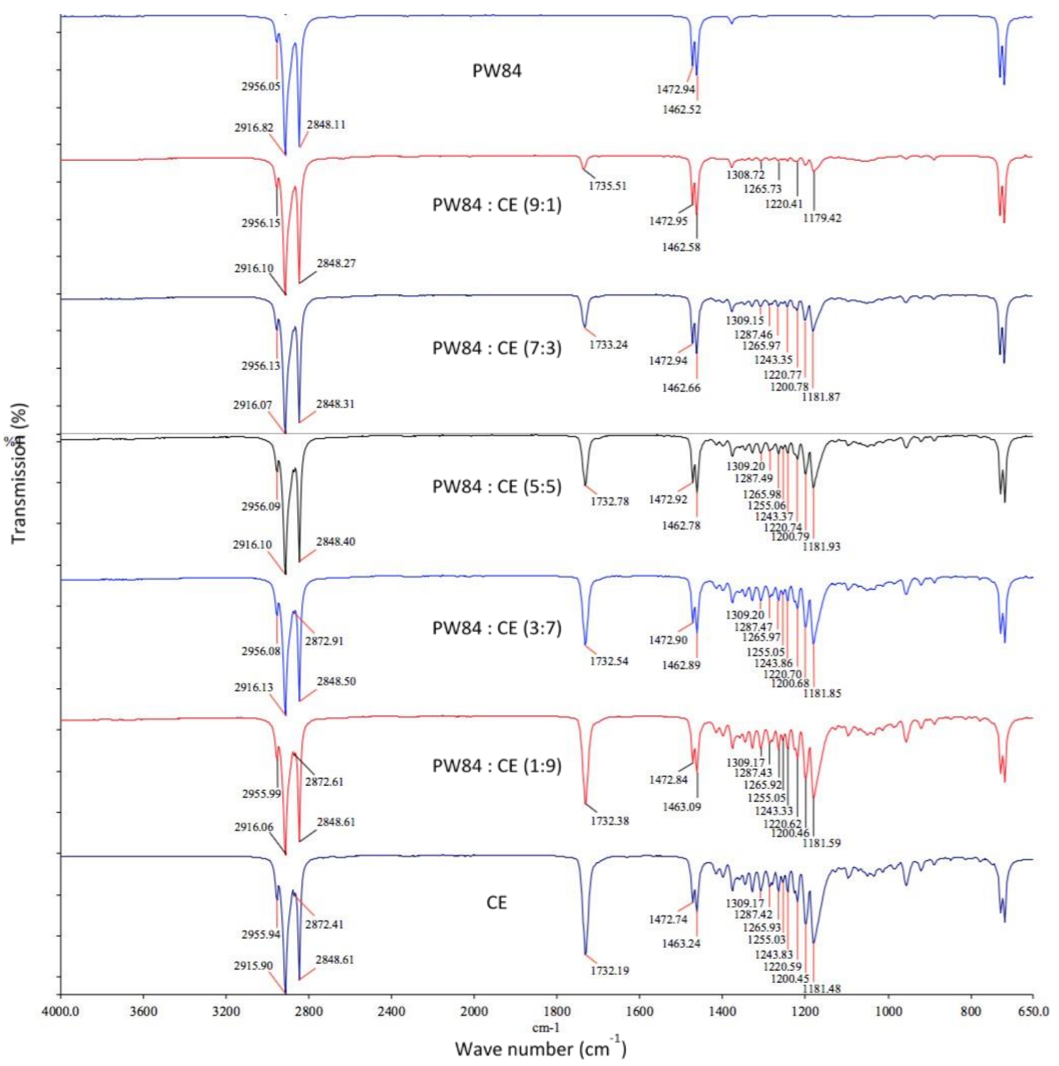

Figure S1. FTIR spectra of (A) PW59, SA and blends; (B) PW84, PW59 and blends; (C) PW84, SA and blends; (D) PW84, CA and blends; (E) PW84, CE and blends (n = 3).

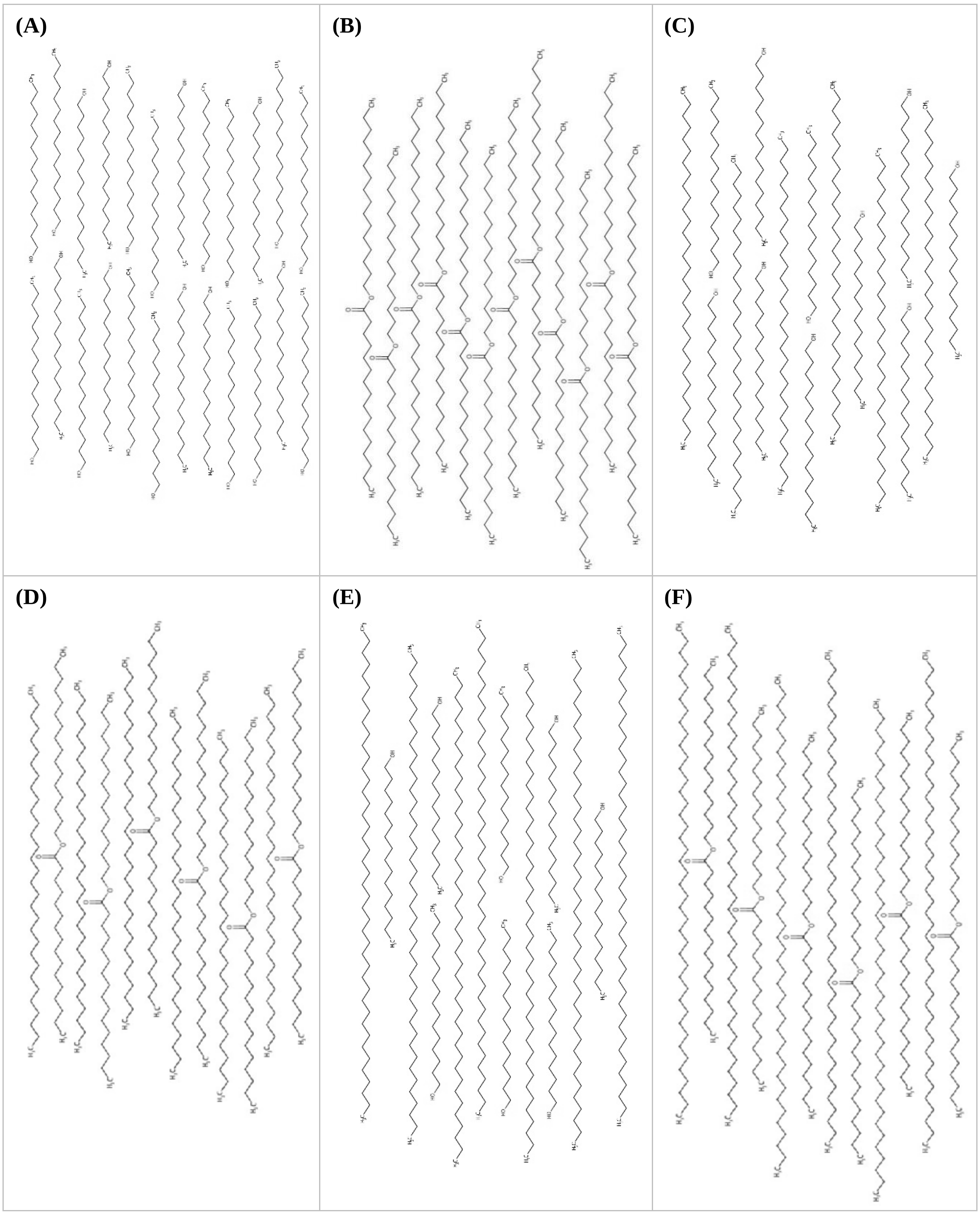

Figure S2. Postulated conformations of (A) CA molecules; (B) CE molecules; (C) PW59 and CA blend; (D) PW59 and CE blend; (E) PW84 and CA blend; (F) PW84 and CE blend under shear, drawn using MarvinSketch program (MarvinSketch 17.1.23.0, ChemAxon, Hungary), which takes into consideration the energy of the conformation of molecules.
